# Deconvoluting the complexity of autophagy in colorectal cancer: From crucial pathways to targeted therapies

**DOI:** 10.3389/fonc.2022.1007509

**Published:** 2022-09-12

**Authors:** Liming Qiang, Hongpeng Li, Zhaohui Wang, Lin Wan, Guangfu Jiang

**Affiliations:** ^1^ Department of Gastroenterology Ward, Guang’an People’s Hospital, Guang’an, China; ^2^ Department of Gastrointestinal Surgery, Guang’an People’s Hospital, Guang’an, China

**Keywords:** colorectal cancer (CRC), autophagy, crucial pathway, therapeutic strategy, targeted therapy

## Abstract

Colorectal cancer (CRC) is a common gastrointestinal tumor with a high degree of malignancy, and most clinical cases are diagnosed at an advanced stage, which has unfortunately missed an opportunity for surgery; therefore, elucidation of the crucial pathways of CRC development and discovery of targeted therapeutic strategies should be anticipated. Autophagy, which is an evolutionarily highly conserved catabolic process, may promote tumorigenesis and development of CRC. On the contrary, autophagy can trigger programmed cell death to inhibit CRC progression. Correspondingly, several targeted therapeutic strategies have been reported in CRC, including small-molecule compounds, polypeptides, non-coding RNAs, photodynamic, and adjuvant therapies. Thus, in this review, we focus on summarizing the crucial pathways of autophagy in CRC, and further discuss the current therapeutic strategies targeting autophagy. Together, these findings may shed light on the key regulatory mechanisms of autophagy and provide more promising therapeutic approaches for the future CRC therapies.

## Graphical Abstract

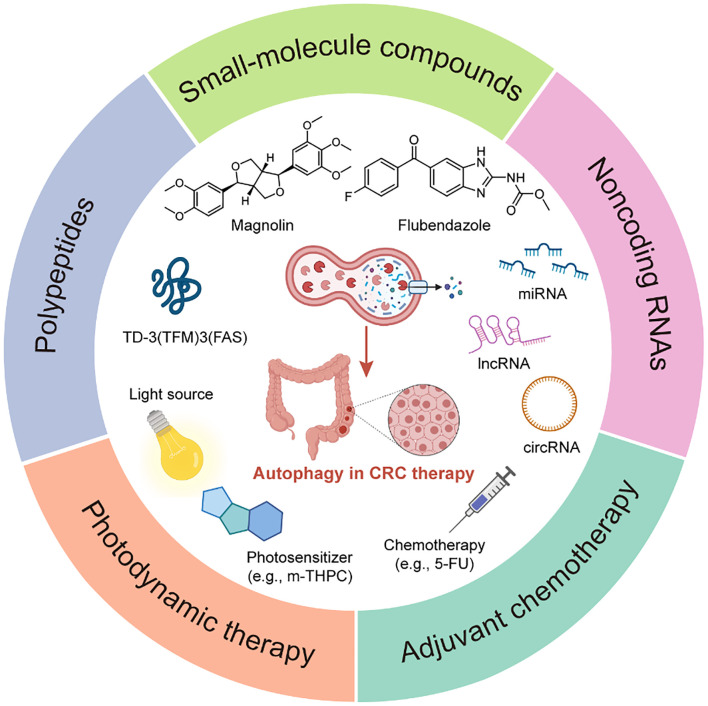

## Introduction

Colorectal cancer (CRC) is well-known as the most common malignant gastrointestinal tumor in the world, with about 1.9 million new cases in 2020, accounting for about 10% of the global cancer incidence, ranking third among all cancers (1). And, CRC mortality constantly remains at a relatively high level, with about 940,000 new deaths in 2020, accounting for 9.4% of global cancer deaths, which is the second leading cause of cancer death followed by lung cancer (1, 2). The 5-year survival rate of CRC patients is approximately 60%-70% with distant stage diagnosis is only 14% (2, 3). Unfortunately, the majority of CRC cases are usually detected in the advanced clinical stage and diagnosed as the distant stage with a poor prognosis (3, 4). Hitherto, the current treatment of CRC has still been limited to the traditional surgery combined with chemoradiotherapy (5); however, chemotherapy drugs, including the most classic 5-Fluorouracil (5-FU), will inevitably develop chemotherapy resistance after long-term treatment, weakening the efficacy and causing tumor recurrence (6). Due to the severe side effects of chemoradiotherapy and the inability of surgical treatment for patients diagnosed at an advanced stage, most patients, especially those with poor prognosis, still lack effective targeted therapy, resulting in a high annual mortality rate of CRC (1, 2, 7). Thus, in-depth exploration of the pathogenesis of CRC and searching for effective targeted therapeutic approaches are urgent to be solved.

Of note, autophagy is an evolutionarily highly conserved catabolic process that regulates the expression of various oncogenes and tumor suppressor genes, which is a double-edged sword in many types of human cancers, such as CRC (8). On one hand, autophagy plays a cytoprotective role by removing misfolded proteins, damaged organelles, and reactive oxygen species, limiting tumorigenesis; on the other hand, autophagy provides energy through catabolism to help tumor cells cope with stress stimuli, such as insufficient oxygen, nutrient deficiencies, or cancer treatment, which leads to tumor progression (9, 10). Currently, there are some small-molecule compounds as autophagic modulators (e.g., chloroquine or hydroxychloroquine) have shown a promising tumor therapeutic potential in clinical trials, and targeting autophagy has gradually been recognized as one of new strategies for potential therapeutic purposes (11). As mentioned above, in this review, we summarize several crucial pathways of autophagy in CRC progression, and further discuss some therapeutic strategies targeting autophagy to improve CRC treatment.

## Crucial pathways for autophagy regulation in CRC

It is well-known that there are five critical stages in the canonical autophagy process, including autophagy initiation, phagophore nucleation, phagophore elongation and maturation, autophagosome and lysosome fusion, autolysosome degradation and recycling (12).

### Autophagy initiation: ULK1-ATG13-FIP200-ATG101

UNC-51-like autophagy-activating kinase 1 (ULK1), as the homologous protein of yeast Atg1, plays a vital role in the autophagy initiation stage. As a conserved promoter of the autophagy process, ULK1 forms ULK complex with autophagy-related gene (ATG) 13, RB1-inducible coiled-coil 1 (RB1CC1; FIP200), and ATG101, which transmit autophagy signals and initiate the formation of autophagosome when they are activated (13). Recently, there is a study has shown that ULK1 is the most frequently mutated gene in The Cancer Genome Atlas (TCGA)-colorectal adenocarcinoma dataset, implying that ULK1 and its regulatory network may be closely related to colorectal carcinogenesis (14). Notably, the upregulation of ULK1 significantly induces autophagy-dependent cell death in RKO human CRC cells, which exerts excellent antiproliferative potency (15).

The mechanistic target of rapamycin (mTOR), the most widely studied negative regulator of autophagy, is usually activated by PI3K-Akt and directly inhibits ULK1 to inhibit autophagy (16). In CRC, FAT tumor suppressor homolog 4 (FAT4) inhibits the PI3K-Akt-mTOR pathway, promoting autophagy and inhibiting migration and invasion in SW480, HCT116 and LOVO human CRC cells (17); similarly, downregulation of pleckstrin homology like domain family A member 2 (PHLDA2) inhibits the PI3K-Akt-mTOR pathway, inducing autophagy and inhibiting proliferation in SW480, HCT116 human CRC cells (18).

**Figure 1:**
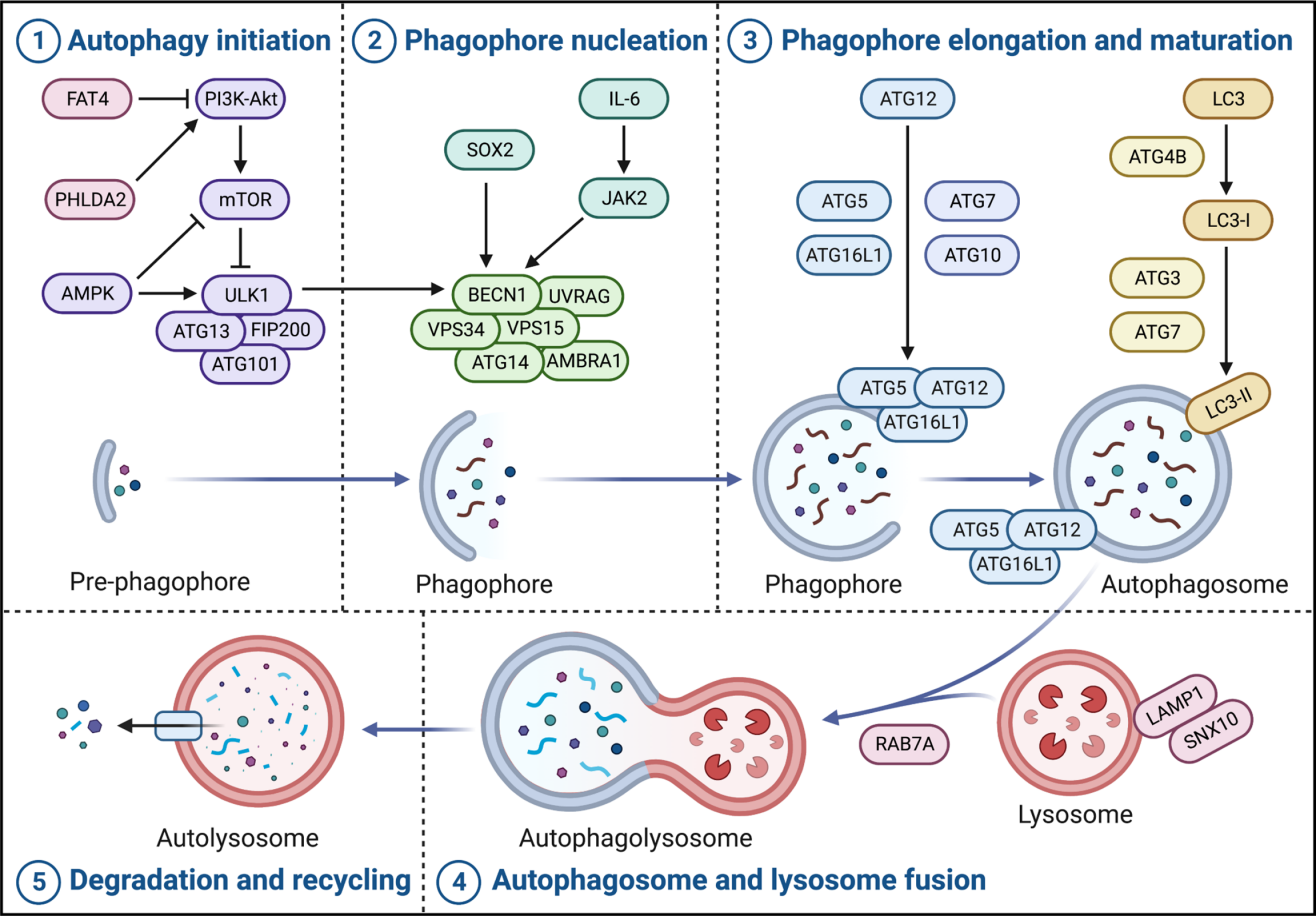
Crucial pathways of autophagy in CRC. Autophagy in CRC broadly contains five stages, namely autophagy initiation, phagophore nucleation, phagophore elongation and maturation, autophagosome and lysosome fusion, and autolysosome degradation and recycling. The ULK complex (ULK1-ATG13-FIP200-ATG101) triggers autophagy initiation and the PI3K complex (BECN1-VPS34-VPS15-ATG14-AMBRA1-UVRAG) promotes phagophore nucleation. Moreover, phagophore elongation and maturation contain two important ubiquitination modifications, one of which is the formation of complexes and localization of ATG5, ATG12, and ATG16L1 to the autophagosome membrane catalyzed by ATG7 and ATG10; the other is the cleavage of LC3 precursor protein by ATG4B and ATG7 to generate LC3-I, and the ATG5-ATG12-ATG16L1 complex catalyzes LC3-I coupling with PE to form LC3-II. Subsequently, RAB7A and LAMP1 are involved in autophagosome and lysosome fusion to form autolysosome. Finally, the contents of the autolysosome are degraded and recycled into the cytoplasm to re-engage in cellular metabolism.

AMP-activated protein kinase (AMPK) is also a critical regulator upstream of ULK1, which directly phosphorylates ULK1 to activate autophagy. In addition, activated AMPK also phosphorylates downstream tuberous sclerosis complex 1 and 2 (TSC1/2) to enhance inhibition of Rheb, thereby inhibiting mTOR and inducing autophagy (19). In HCT116 and HT29 human CRC cells, knockdown of AMPK restricted tumor autophagy-dependent cell death, leading to tumor progression (20) (**Figure 1**).

### Phagophore nucleation: BECN1-VPS34-VPS15-ATG14-AMBRA1-UVRAG

Coiled-coil myosin-like BCL2-interacting protein (BECN1), as the homologous protein of yeast Atg6, forms the class III phosphoinositide 3-kinase (PI3K) complex with phosphatidylinositol 3-kinase catalytic subunit type 3 (PIK3C3; VPS34), phosphoinositide 3-kinase regulatory subunit 4 (PIK3R4; VPS15), ATG14, autophagy and beclin 1 regulator 1 (AMBRA1) and UV radiation resistance-associated (UVRAG), which is responsible for the phagophore nucleation; specifically, it is usually phosphorylated by activated ULK1 and acts as an integral scaffold for the PI3K complex, recruiting autophagy-related proteins such as ATG9 to localize to phagophore (21). Interestingly, a recent study has shown that the transcription factor sex-determining region Y-box2 (SOX2) can bind to the promoter of BECN1 to induce its transcription and activate autophagy in SW480 and SW620 human CRC cells, resulting in tumor progression (22); and, interleukin-6 (IL-6), independently of its substrate signal transducer and activator of transcription 3 (STAT3), induces Janus kinase 2 (JAK2) to phosphorylate the tyrosine residue at position 333 of BECN1 in LoVo human CRC cells, enhancing BECN1-VPS34 interaction and autophagy significantly, which leading to a poor prognosis of CRC patients (23). In addition, the inhibition of VPS34 also strongly reduces the level of autophagy in Caco-2 human CRC cells (24) (**Figure 1**).

### Phagophore elongation and maturation: ATG5-ATG12-ATG16L1/ATG4B-ATG7-LC3

There are two important ubiquitination modifications in phagophore elongation and maturation, one of which is that ATG5, ATG12 and ATG16L1 are catalyzed by ATG7 and ATG10 to form a complex and localize to the autophagosome membrane (25). In CRC, patients with high ATG5 expression generally have a poorer prognosis and are more likely to cause tumor recurrence (26). Interestingly, the *ATG16L1* ^T300A^ variant elicits a defect of autophagy, which is closely associated with better patient prognosis (27). Notably, ATG16L2, as a paralog of ATG16L1, which N-terminal region also binds to the ATG5-ATG12 complex like ATG16L1, but is not recruited to the autophagosome membrane, possibly acting as a potential competitive ATG16L1 inhibitor to inhibit autophagy; in CRC, its overexpression inhibits tumor proliferation *in vitro* and *in vivo*, and is associated with usually a long survival rate (28). The other ubiquitination modification is that ATG4B and ATG7 cleave the LC3 precursor protein to generate LC3-I, and the ATG5-ATG12-ATG16L1 complex catalyzes the coupling of LC3-I to phosphatidylethanolamine (PE) to form LC3-II (MAP1LC3B) (29). In HCT116 and Caco2 human CRC cells, silencing of ATG4B increases autophagy levels (30). ATG7 is usually highly expressed in CRC cells and has nothing to do with the survival of intestinal epithelial cells, but affects the survival of tumors, which is a potential target in CRC (31) (**Figure 1**).

### Autophagosome and lysosome fusion, autolysosome degradation and recycling

Ras-related protein Rab-7a (RAB7A) is a critical small GTPase that promotes autophagosome-lysosome fusion, which together with lysosome-associated membrane protein 1 (LAMP1), participates in autophagosome-lysosome fusion to form autolysosome. Subsequently, the contents of the autolysosome are degraded and recycled into the cytoplasm to re-engage in cellular metabolism (25). Currently, sorting nexin 10 (SNX10) has been reported to interact with ATG12-ATG5 conjugates and LAMP1 to promote autophagosome-lysosome fusion, inhibiting the occurrence and progression of CRC (32) (**Figure 1**).

## Therapeutic strategies targeting autophagy for CRC therapy

Currently, the therapeutic strategies for CRC are mainly traditional tumor treatment approaches, namely surgery, radiotherapy, and chemotherapy. However, for patients whose conditions are not suitable for conventional treatment approaches, such as patients with recurrent, metastatic tumors or locally advanced inoperable treatment, targeting autophagy with small-molecule compounds has shown solid therapeutic potential. Notably, some emerging therapeutic strategies such as polypeptide and non-coding RNAs (ncRNAs) that modulate autophagy for CRC therapy also have achieved promising preclinical results. Additionally, autophagy-targeting of photodynamic therapy and autophagy adjuvant chemotherapy strategies demonstrate the broad prospects of autophagy in CRC therapy.

### Small-molecule compounds targeting autophagy

**Figure 2:**
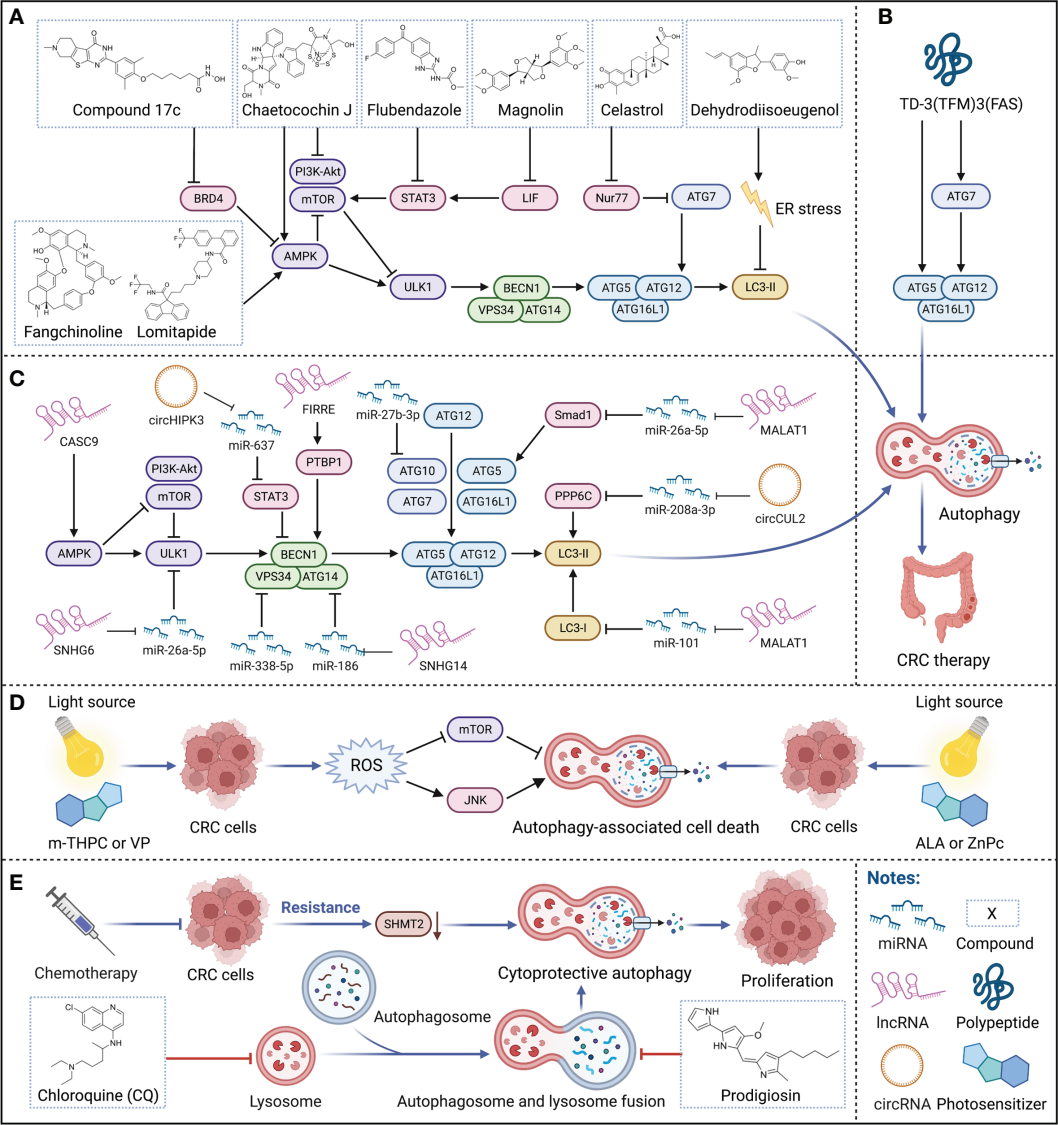
Therapeutic strategies targeting autophagy for CRC therapy. **(A–C)** Small-molecule compounds, polypeptide, and ncRNAs for targeting autophagy in CRC therapy. Various small-molecule compounds, the polypeptide, and multiple ncRNAs modulate critical regulators of autophagy to treat CRC. **(D)** Photodynamic therapy targeting autophagy in CRC therapy. Multiple photosensitizers with specific wavelength light source irradiation induce autophagy-associated cell death, participating in CRC therapy. **(E)** Adjuvant chemotherapy by autophagy for CRC therapy. Autophagy inhibitors can effectively suppress the cytoprotective autophagy triggered by long-term chemotherapy and restore the sensitivity of tumors to chemotherapy drugs, enhancing the effectiveness of chemotherapy.

Hitherto, a variety of small-molecule compounds have demonstrated compelling efficacy in CRC therapy *via* modulating autophagy. For instance, Fangchinoline, an alkaloid monomer with anti-inflammatory activity derived from the *Stephaniae tetrandrine* S. Moore, is a novel autophagy agonist in CRC that activates the AMPK-mTOR-ULK1 pathway to induce autophagy-dependent cell death in HT29 and HCT116 human CRC cells and also exerts an effective growth inhibition of tumors *in vivo* (33). Similarly, Chaetocochin J, an alkaloid monomer derived from *Chaetomium* sp, activates AMPK and inhibits PI3K-Akt-mTOR to induce autophagy, manifesting a solid antiproliferative effect in RKO, HCT116 and SW480 human CRC cells with IC_50_s of 0.56, 0.61 and 0.65 μM (34). Celastrol, contained in *Tripterygium wilfordii*, inhibits the transcription factor Nur77 and upregulates ATG7 to induce autophagy, achieving favorable antitumor effects in HCT116 and SW480 human CRC cells and the HCT116 xenograft mouse model (35). Magnolin, a lignan monomer with anti-inflammatory and antioxidant activity derived from *Magnolia biondii*, significantly upregulates LC-3B and downregulates p62 by inhibiting leukemia inhibitory factor (LIF)- STAT3-Mcl-1 to induce autophagy in HCT116 and SW480 human CRC cells and HCT116 xenograft model, which showing excellent anticancer potential (36). Notably, Dehydrodiisoeugenol, a traditional Chinese medicine monomer composition derived from nutmeg, inhibits the late stage of autophagy by inducing endoplasmic reticulum (ER) stress, which greatly restricts the growth and proliferation of tumors; it exhibits convincing antiproliferative activity not only in HCT116 and SW620 human CRC cells with IC_50_s of 54.32μM and 46.74μM but also in cell-derived xenograft (CDX) and patient-derived tumor xenograft (PDX) models with lower toxicity (37). In addition to natural products, there are several repositioning small-molecule compounds that contribute to CRC therapy *via* modulating autophagy. For instance, lomitapide, a clinical drug approved by the Food and Drug Administration (FDA) for the treatment of hypercholesterolemia, is recently reported to upregulate AMPK phosphorylation and promote the formation of BECN1-VPS34-ATG14 complex, thereby inducing autophagy in HCT116 and HT29 human CRC cells, which significantly inhibits tumor proliferation *in vitro* and *in vivo* (20). Similarly, flubendazole, an anthelmintic drug approved by FDA, downregulates STAT3 phosphorylation levels, mTOR and p62, and upregulates Beclin 1 and LC3-I/II in HCT116, RKO and SW480 human CRC cells, which promoting the initiation of autophagy to prevent tumor progression without substantially affecting normal cell proliferation (38). Currently, a series of dual-target inhibitors of bromodomain-containing protein 4 (BRD4) and histone deacetylases (HDAC) based on the structure design and optimization have been reported, among which compound 17c is the most potent inhibitor of BRD4 and HDAC, induces autophagy *via* BRD4-AMPK-mTOR-ULK1 pathway, showing promising antiproliferative activities *in vitro* and *in vivo* (39) (**Figure 2A**).

### Polypeptides targeting autophagy

Notably, compared with small-molecule compounds, polypeptides usually have higher selectivity and stability *in vivo* with a low probability of immune system rejection and are expected to achieve higher efficacy. Recently, TD-3(TFM)3(FAS), a novel DNA tetrahedron (TD) with two types of therapeutic peptides (FAS peptides and FK-16 peptides) has been designed; among them, FK-16 is delivered to the cytoplasm of HT-29 human CRC cells by cell-penetrating peptide, further upregulating the expression of p53, ATG5, and ATG7, inducing autophagy-dependent cell death and exerting strong and specific tumor-suppressive efficacy (40) (**Figure 2B**). In addition, LL-37, an antimicrobial peptide, is closely related to cellular processes such as apoptosis and autophagy, inhibiting the carcinogenesis of intestinal cells. However, due to the complex pathogenesis of CRC, whether LL-37 can treat CRC by regulating autophagy still need to explore (41).

### Noncoding RNAs targeting autophagy

In recent years, ncRNA has been found to play critical roles in various cellular physiological processes and is closely related to the occurrence and progression of diseases, especially cancer. Currently, there are a series of regulatory strategies have been applied to tumor diagnosis and clinical trials (42). Notably, autophagy regulated by ncRNAs can affect multiple core processes involved in tumor survival, including proliferation, apoptosis, invasion, and metastasis (43). Therefore, in-depth exploration of the mechanism of ncRNAs regulating autophagy is expected to provide new directions for CRC therapy.

Of note, microRNAs (miRNAs) are short ncRNAs that are extensively studied in cancer due to their modulation of various downstream mRNAs, which have been reported as novel specific biomarkers in multiple cancers. For instance, miR-338-5p is often highly expressed in the more malignant CRC phenotype, implying that it could serve as a promising potential biomarker for CRC diagnosis; it inhibits PIK3C3 and suppresses autophagy to promote tumor invasion and migration (44). Similarly, miR-27b-3p has also been reported as a potential therapeutic target for CRC that suppresses autophagy by inhibiting ATG10, which also helps reverse the resistance developed by long-term chemotherapy (45) (**Figure 2C**).

Notably, long non-coding RNAs (lncRNAs) modulate various biological processes, which are closely related to the occurrence and development of multiple diseases, and as a current research hotspot in cancer pathology. Metastasis-associated lung adenocarcinoma transcript 1 (MALAT1), one of the first lncRNAs reported to be involved in cancer metastasis, is aberrantly expressed in multiple human malignancies and can act as a sponge for various miRNAs. In CRC, MALAT1 acts as a sponge for miR-101 to activate autophagy, promoting proliferation and inhibiting apoptosis in HCT116 and SW620 human CRC cells; therefore, the high expression of MALAT1 is closely related to poor prognosis in CRC patients (46). Interestingly, MALAT1 can also act as a sponge for miR-26a-5p, reversing the inhibition of Smad1 by miR-26a-5p to elicit Smad1 upregulation; Smad1 can bind to the ATG5 promoter, induce the transcription of ATG5 to activate autophagy, promoting proliferation and metastasis in HT29 and SW1116 human CRC cells (47). Similarly, lncRNA small nucleolar RNA host gene 6 (SNHG6) acts as a sponge for miR-26a-5p, upregulating ULK1 and activating autophagy in RKO, HT29 and HCT116 human CRC cells (48). In addition, lncRNA small nucleolar RNA host gene 14 (SNHG14) is often highly expressed in a variety of cancers, leading to poor progression; in CRC, SNHG14 suppresses miR-186 to upregulate ATG14, inducing autophagy in SW620 and SW480 human CRC cells (49). Cancer susceptibility candidate 9 (CASC9) is a lncRNA highly expressed in CRC in both TCGA and The Encyclopedia of RNA Interactomes (ENCORI) datasets, which high expression is associated with poor patient prognosis. Inhibition of CASC9 upregulates the phosphorylation level of AMPK and suppresses Akt-mTOR signaling, inhibiting tumor growth and inducing autophagy in HCT116 and SW480 human CRC cells, which is a promising strategy for CRC therapy (50). Moreover, lncRNA functional intergenic repeating RNA element (FIRRE) is often located in the nucleus and can bind PTBP1 to promote its translocation to the cytoplasm to stabilize the cytoplasmic mRNA BECN1, increasing the level of autophagy in RKO human CRC cells (51) (**Figure 2C**).

Importantly, circular RNAs (circRNAs) are a special class of ncRNAs that often function as competing endogenous RNAs (ceRNAs) for miRNAs, which implied an emerging strategy in cancer therapy. For instance, circCUL2 acts as a sponge for miR-208a-3p to inhibit tumor proliferation *via* upregulating protein phosphatase 6 catalytic subunit (PPP6C), which induces autophagy in SW480 and SW620 human CRC cells and the SW480 xenograft model (52). Similarly, circHIPK3 acts as a sponge for miR-637 to upregulate STAT3, activating downstream Bcl-2 to suppress beclin1 to inhibit autophagy *in vitro* and *in vivo* (53) (**Figure 2C**). Although the application of ncRNAs in tumor diagnosis and treatment is still in the early stage, as more and more mechanisms are discovered, we believe that the clinical application of ncRNAs is just around the corner.

### Photodynamic therapy targeting autophagy

Photodynamic therapy (PDT), an emerging minimally invasive procedure for cancer therapy, relies on specific wavelength light source irradiation to activate photosensitizers in tumors to generate biotoxic singlet oxygen and other highly reactive oxygen species (ROS), which in turn oxidatively damages tumors, and exerts therapeutic effects by inducing various forms of cell death such as apoptosis or autophagy (54, 55). Compared with traditional treatment strategies for cancer, PDT is less invasive, has better selectivity and a broader range of applications, and can be repeated multiple times without drug resistance or toxicity (55). Currently, PDT to induce autophagy has achieved a series of progress in CRC therapy. For instance, treatment of HCT116 and SW480 human CRC cells with the second-generation photosensitizers meta-tetrahydroxyphenylchlorin (m-THPC) and verteporfin (VP) produces a large amount of ROS, induces autophagy by activating c-Jun N-terminal kinase (JNK) and inhibiting the phosphorylation of mTOR; in addition, m-THPC and VP also effectively suppress the tumor progression of HCT116 xenografts (56). Importantly, both high-speed and short-term acute PDT (aPDT) and low-speed and long-term metronomic PDT (mPDT) with the photosensitizer 5-aminolevulinic acid (ALA) can induce autophagy in SW837 human CRC cells, and ALA-mPDT induces autophagy earlier and exhibits stronger antitumor effect than ALA-aPDT (57). Similarly, PDT with the photosensitizer zinc phthalocyanine (ZnPc) at a light flux of 12 J/cm^2^ or 24 J/cm^2^ induces autophagy in SW480 human CRC cells (58) (**Figure 2D**).

### Adjuvant chemotherapy targeting autophagy

5-FU is the first drug recognized as an effective chemotherapy drug for CRC, which has been widely used in CRC clinical therapy since 1957. Unfortunately, long-term treatment inevitably develops chemoresistance, resulting in tumor relapse; notably, autophagy plays a critical role in its drug resistance mechanism. Once 5-FU gives tumors stress, autophagy can provide additional nutrients to meet the metabolic needs of tumors, resulting in abnormal proliferation and weakening the effectiveness of chemotherapy (6). Therefore, the therapeutic strategy of 5-FU combined with autophagy inhibition may considerably improve the survival rate of CRC patients. For instance, the treatment of 5-FU decreases the expression of serine hydroxymethyltransferase-2 (SHMT2), which promotes autophagy and triggers 5-FU resistance, resulting in poor prognosis of CRC patients; while 5-FU combined with the autophagy inhibitor chloroquine (CQ) reverses the insensitivity to 5-FU in CRC cells with low SHMT2 expression *in vitro* and *in vivo*, thereby enhancing the effect of chemotherapy (59). Prodigiosin, a secondary metabolite synthesized by bacteria such as *Serratia marcescens*, inhibits autophagy by blocking the fusion of autophagosome and lysosome and suppressing the activity of lysosomal hydrolase, further triggers the accumulation of LC3B-II and SQSTM, enhances the sensitivity of tumors to 5-FU, synergizing with 5-FU to inhibit CRC progression in HCT116 and SW480 human CRC cells and HCT116 cells nude mice xenografts (60). In addition, knockdown of ATG7 resensitizes SW480 and HT29 human CRC cells to Irinotecan and 5-FU, overcoming chemoresistance, again confirming the feasibility of autophagy inhibitors to alleviate chemoresistance (31). Similarly, inhibition of autophagy also increases radiosensitivity, which implies a potential CRC therapeutic strategy. For instance, downregulation of long non-coding RNA homeobox transcript antisense intergenic RNA (HOTAIR) upregulates miR-93 to downregulate ATG12, improving the effect of radiotherapy *via* inhibiting autophagy in SW480 and HCT116 human CRC cells and CRC xenograft models (61) (**Figure 2E**).

## Conclusions and perspectives

CRC is a common malignant tumor with a high global incidence and mortality rate, and its therapeutic strategies have been mainly dependent on surgery, radiotherapy, and chemotherapy so far. Unfortunately, some patients are already at an advanced stage of CRC when they are detected and cannot be treated surgically. Therefore, development of some new emerging therapeutic approaches should be urgent. On one hand, autophagy removes damaged nucleic acids and cellular organelles to protect cells from stress damage, effectively reducing the probability of CRC occurrence. On the other hand, autophagy may help CRC respond to the therapeutic stimuli, provides the energy required for tumor proliferation through catabolism, and even resists drug stimuli to resistance.

Notably, the crucial pathway for autophagy regulation is one of the current research hotspots in CRC pathology. Many canonical regulators of autophagy processes (e.g., ULK1, ATG5, ATG16L1) are aberrantly expressed in CRC patients. In addition, some studies knocked down or overexpressed critical regulators in each stage of autophagy to rationally utilize its two sides, initially showing favorable antitumor effects. In short, an in-depth exploration of crucial pathways for autophagy regulation in CRC and the corresponding effective interventions will considerably improve CRC therapy.

Currently, several small-molecule compounds targeting autophagy (e.g., Fangchinoline, Chaetocochin J, and Celastrol) have been achieving some promising preclinical results and exerted a great potential on potential CRC therapies. Notably, compared with small-molecule compounds, polypeptides [e.g., TD-3(TFM)3(FAS)] generally have higher selectivity and stability *in vivo*, and are less susceptible to rejection by the immune system, exhibiting high therapeutic potential for CRC. In addition, some key regulatory factors (e.g., microRNA miR-338-5p, lncRNA MALAT1, and circRNA circCUL2) have also been continuously identified as autophagy-related biomarkers in CRC, providing accumulating evidence for the availability of clinical diagnosis and treatment. As a new emerging therapeutic strategy, photodynamic therapy also achieved inspiring stage results in CRC therapy by inducing autophagy, which provides more options for CRC patients. Interestingly, autophagy reverses long-term chemotherapy-induced resistance and sensitize tumors to chemotherapeutic drugs.

In summary, modulating autophagy has been emerging as a promising strategy for CRC therapy, which can benefit the patients who are not suitable for traditional treatment, and can be used as adjuvant chemotherapy to overcome drug resistance. Importantly, the rapid development of new technologies, such as artificial intelligence (AI) seems to be delineated the intricate dynamic balance of autophagy between CRC progression and treatment (62). With the continuous exploration of the relationship between autophagy and CRC, we believe that the Janus roles of autophagy would be subtly manipulated, and more effectively therapeutic strategies will be exploited to greatly improve potential CRC therapies in the future.

## Author contributions

LQ, HL participates in manuscript writing and figure drawing; ZW, LW participates in references collection and manuscript formatting adjustments. GJ reviewed and edited the manuscript. All the authors approved the submitted version.

## Funding

This work was supported by grants from the fund of the high-quality development of Guang ‘an People’s Hospital (Grant No. 21FZ008).

## Conflict of interest

The authors declare that the research was conducted in the absence of any commercial or financial relationships that could be construed as a potential conflict of interest.

## Publisher’s note

All claims expressed in this article are solely those of the authors and do not necessarily represent those of their affiliated organizations, or those of the publisher, the editors and the reviewers. Any product that may be evaluated in this article, or claim that may be made by its manufacturer, is not guaranteed or endorsed by the publisher.

## Glossary

**Table:**

5-FU	5-Fluorouracil
AI	artificial intelligence
ALA	5-aminolevulinic acid
AMBRA1	autophagy and beclin 1 regulator 1
AMPK	AMP-activated protein kinase
aPDT	acute PDT
ATG	autophagy-related gene
BECN1	coiled-coil myosin-like BCL2-interacting protein
BRD4	bromodomain-containing protein 4
CASC9	cancer susceptibility candidate 9
CDX	cell-derived xenograft
ceRNA	competing endogenous RNA
circRNA	circular RNA
CQ	chloroquine
CRC	colorectal cancer
ENCORI	The Encyclopedia of RNA Interactomes
ER	endoplasmic reticulum
FAT4	FAT tumor suppressor homolog 4
FDA	Food and Drug Administration
FIRRE	functional intergenic repeating RNA element
HDAC	histone deacetylases
HOTAIR	homeobox transcript antisense intergenic RNA
IL-6	interleukin-6
JAK2	Janus kinase 2
JNK	c-Jun N-terminal kinase
LAMP1	lysosome-associated membrane protein 1
LIF	leukemia inhibitory factor
lncRNA	long non-coding RNA
MALAT1	metastasis-associated lung adenocarcinoma transcript 1
miRNAs	microRNAs
mPDT	metronomic PDT
mTOR	mechanistic target of rapamycin
m-THPC	meta-tetrahydroxyphenylchlorin
ncRNA	non-coding RNA
PDT	photodynamic therapy
PDX	patient-derived tumor xenograft
PE	phosphatidylethanolamine
PHLDA2	pleckstrin homology like domain family A member 2
PI3K	phosphoinositide 3-kinase
PIK3C3	phosphatidylinositol 3-kinase catalytic subunit type 3
PIK3R4	phosphoinositide 3-kinase regulatory subunit 4
PPP6C	protein phosphatase 6 catalytic subunit
RAB7A	Ras-related protein Rab-7a
RB1CC1	RB1-inducible coiled-coil 1
ROS	reactive oxygen species
SHMT2	serine hydroxymethyltransferase-2
SNHG6	small nucleolar RNA host gene 6
SNHG14	small nucleolar RNA host gene 14
SNX10	sorting nexin 10
SOX2	sex-determining region Y-box2
STAT3	signal transducer and activator of transcription 3
TCGA	The Cancer Genome Atlas
TD	tetrahedron
TSC1/2	tuberous sclerosis complex 1 and 2
ULK1	UNC-51-like autophagy-activating kinase 1
UVRAG	UV radiation resistance-associated
VP	verteporfin
ZnPc	zinc phthalocyanine.
